# Predicting Protein Model Quality from Sequence Alignments by Support Vector Machines

**DOI:** 10.4172/jpb.S9-001

**Published:** 2013-11-04

**Authors:** Xin Deng, Jilong Li, Jianlin Cheng

**Affiliations:** 1 Computer Science Department, University of Missouri-Columbia, Columbia, MO, USA; 2 Informatics Institute, University of Missouri-Columbia, Columbia, MO, USA; 3 C. Bond Life Science Center, University of Missouri-Columbia, Columbia, MO, USA

**Keywords:** Protein structure model, Protein structure prediction, Protein model quality, Sequence alignment, Support vector machine

## Abstract

Assessing the quality of a protein structure model is essential for protein structure prediction. Here, we developed a Support Vector Machine (SVM) method to predict the quality score (GDT-TS score) of a protein structure model from the features extracted from the sequence alignment used to generate the model. We developed a Support Vector Machine (SVM) model quality assessment method, taking either a query-single-template pairwise alignment or a query-multitemplate alignment as input. For the pairwise alignment scheme, the input features fed into the SVM predictor include the normalized e-value of the given alignment, the percentage of identical residue pairs in the alignment, the percentage of residues of the query aligned with those of the template, and the sum of the BLOSUM scores of all aligned residues divided by the length of the aligned positions. Similarly, for the multiple-alignment scheme, the input features include the percentage of the residues of the target sequence aligned with those in one or more templates, the percentage of aligned residues of the target sequence that are the same as that of any one template, the average BLOSUM score of aligned residues and the average Gonnet160 score of aligned residues. A SVM regression predictor was trained on the training data to predict the GDT-TS scores of the models from the input features. The Root Mean Square Error (RMSE) and the Absolute Mean Error (ABS) between predicted and real GDT-TS scores were calculated to evaluate the performance. A five-fold cross validation was applied to select the best parameter values based on the average RMSE and ABS on the five folds. The RMSE and ABS of the optimized SVM predictor on the testing data were close to 0.1. The good performance of the SVM and sequence alignment based predictor indicates that integrating sequence alignment features with a SVM is effective for protein model quality assessment.

## Background

The knowledge of protein three-dimensional (3D) structures is vitally important for biomedical research, such as protein function analysis, mutagenesis experiments and rational drug design. Although the X-ray crystallography technique can determine protein 3D structures with high resolution, they are still time consuming, expensive and cannot be readily applied to the proteins that cannot be successfully crystallized, including most membrane proteins. The nuclear magnetic resonance (NMR) is a powerful tool that can determine the 3D structures of membrane proteins of small and medium size in solutions [1-3], but it is also time-consuming and costly. In order to acquire the protein structural information at a large scale and in a timely manner, high throughput fast computational protein structure prediction methods, such as homology modelling [4,5], need to be used. Since the accuracy of predicted protein structures depend on the relatedness of homologous structural templates and the correctness of sequence alignment [4], assessing the quality of protein structural models is important for controlling and analysing the quality of the predicted models.

Thus, protein model quality assessment plays a profound role in protein structure prediction and related applications [6]. Accurate quality assessment of protein models can help rank a pool of candidate models predicted for a given query protein. A number of model quality assessment methods and tools, such as ModelEvaluator [7], APOLLO [8], QMEAN [9], have been developed. These methods evaluate the quality of models based on the structural information extracted from protein models, without considering the source information (e.g. sequence alignment, homologous template structure), used to generate the models. The quality assessment methods without utilizing the source information may be considered a black box approach, while those considering the source information [10], is a white box approach [11].

Since the factors of largely determining the quality of a model, such as the sequence similarity between a query protein and a homologous template structure are generally available in the template-based protein structure prediction (e.g. homology modelling and fold recognition), the white box approach can take advantage of the information to improve model quality assessment.

Here, extending from our previous model quality assessment method based on a query-single-template alignment [12], we designed and developed a support vector machine (SVM) [13] and alignment-based model quality assessment method, taking either a query-single template pairwise alignment or a query-multi template alignment as input to predict the GDT-TS score of a model generated from the input alignment. The method can be applied to select the protein models based on the query template alignments used to generate the models in the widely used template-based protein modelling process.

## Methods

**Figure 1:**
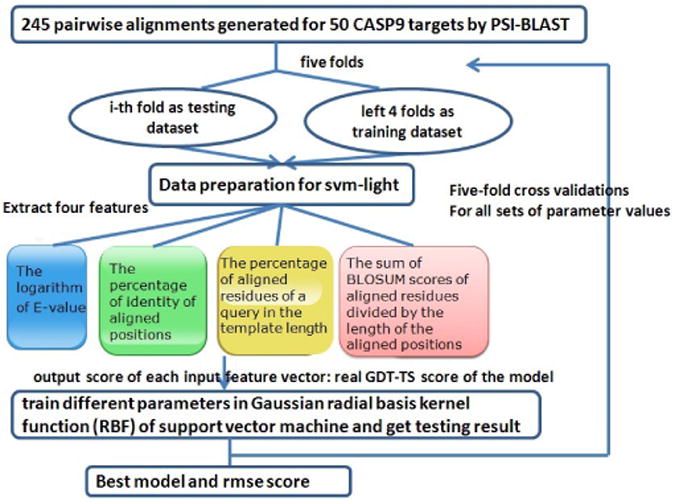
The workflow of the pairwise alignment based SVM model quality prediction method.

Figure 1 shows the workflow how the SVM model quality assessment method uses the features extracted from a query-single-template pairwise alignment to predict model quality. The input features provided to the SVM predictor include the logarithm of e-value of the query template alignment, the percent of identical residue pairs in aligned positions, the percent of residues of the query that are aligned with a residue in the template and the average of BLOSUM [14] scores of all aligned residue pairs. The input feature vectors in the training data set were extracted from 245 pairwise protein sequence alignments generated for 50 CASP9 (the 9th Critical Assessment of Techniques for Protein Structure Prediction [15]) targets by PSI-BLAST [16]. The output score of each input feature vector was the real GDT-TS [17] score of the model generated from the corresponding pairwise alignment. The real GDT-TS score is the structural similarity score between a model and its corresponding native structure calculated by the TM-score program [18]. This data was used to train a SVM regression predictor equipped with a Gaussian radial basis kernel (RBF) to predict the GDT-TS scores of models from the input features. The SVM-Light software package [19] was employed to carry out the training and testing experiments. Three parameters of the SVM, including the epsilon width of the regression tube (w), the margin option (c) and the gamma in the RBF kernel (g) were tuned during the training process. The root mean square error (RMSE) and the absolute mean error (ABS) between the predicted and real GDT-TS scores were used as the evaluation scheme to optimize the parameter values. Three standard crossvalidation methods are commonly adopted to check the effectiveness of a predictor, including independent dataset test, K-fold cross-validation and jackknife test [20]. Here, we utilized the five-fold cross validation approach as many other SVM based prediction methods do in order to achieve higher computational efficiency. Specifically, many rounds of five-fold cross validations were applied to the training data to select the best parameter values of w from 0.5, 0.2, 0.1, 0.05, 0.02 and 0.01 and c from 2.0, 1.0, 0.5, 0.1, 0.05 and 0.01 and g from 0.5, 0.3, 0.2, 0.1, 0.05, 0.01, 0.005 and 0.001, in order to reduce the average ABS and RMSE on all the five folds. The set of parameter values with the lowest RMSE and ABS was selected.

**Figure 2:**
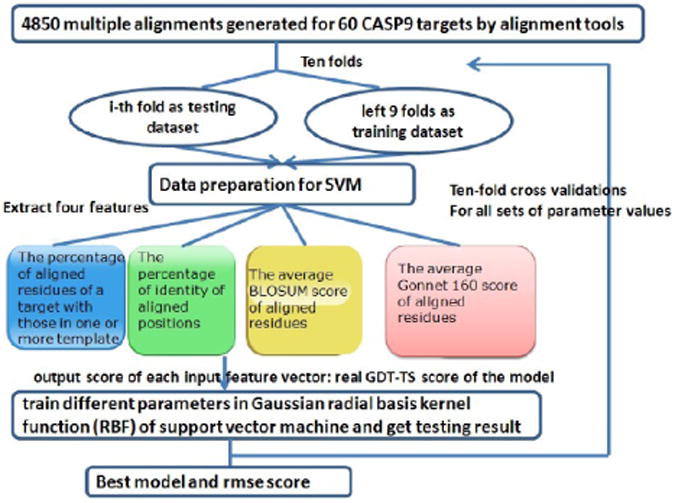
The workflow of the multiple alignment based SVM model quality prediction method.

Similarly, Figure 2 shows the workflow of the SVM model quality assessment method based on the features extracted from the query-multi template alignment used to generate the model. The input features include the percentage of the residues of the target sequence aligned with those in one or more templates, the percentage of identical residues of the target sequence that are the same as that of any one template, the average BLOSUM score of aligned residues, and the average Gonnet160 score [21] of aligned residues. Specifically, as for the average BLOSUM score, if a residue of the target is aligned with those in multiple templates, the BLOSUM score between the residue of the target and that of the template ranked higher in the alignment file (e.g. more significant) is counted. Consequently, the average BLOSUM score associated with all aligned residues of the target sequence was calculated as one feature. The average Gonnet 160 score of all aligned residues is calculated in a similar way. The input feature vectors in the training data set were extracted from 4850 multiple protein sequence alignments generated for 60 CASP9 targets by different alignment tools, such as BLAST, PSI-BLAST [16], HHSearch [22], SAM [23], and SPEM [24], and the output score of each input feature vector was the real GDT-TS score of the model generated from the corresponding multiple alignment. Many rounds of ten-fold cross validations were applied to the training data to select the best parameter values of w from 0.1, 0.08, 0.06, 0.05, 0.02 and 0.01 and g from 0.5, 0.4, 0.3, 0.2, 0.1, 0.05, 0.01, 0.005 and 0.001 and c from 2.0, 1.0, 0.5, 0.1, 0.05 and 0.01.

## Results

### Evaluation of the pairwise alignment based SVM model quality assessment method

The global average RMSE and ABS of the SVM trained with the best set of parameter values (w, c, g)=(0.02, 1.0, 0.5) on the five-fold training data set were 0.083 and 0.061, respectively. The trained pairwise alignment based SVM predictor was applied to predict the GDT-TS scores of models of 46 CASP9 targets generated from 225 PSI-BLAST alignments that were not used in training. The RMSE and ABS were respectively 0.098 and 0.073, demonstrating that the predicted GDT-TS scores are close to the real ones. The RMSE and ABS of the trained SVM with the best parameter set on each fold of the training data, as well as the testing data set are shown in Table 1.

Moreover, we used the predicted model quality scores to rank the models of 46 CASP9 targets [11]. The total real GDT-TS score of the top 1 models selected by the SVM predictor for these targets was compared with that of the top 1 models selected, according to the e-values (i.e. significance) of the PSI-BLAST alignments and that of the top 1 models selected by APOLLO [8], a black box quality assessment tool using a pairwise model comparison approach. The total GDT-TS score of the models selected by the SVM predictor is 20.95, which is higher than 20.10 of the pure e-value based model selection method, as well as 19.53 of APOLLO [8]. The ttest and Wilcox-test were respectively performed, in order to calculate the p-values on the scores of our SVM predictor and the e-value based model selection method, as well as on the scores of our SVM predictor and the APOLLO method. The p-values are reported in Table 2. The results suggest the SVM predictor based on pairwise alignments performed significantly better than the e-value based predictor and APOLLO, according to the standard pvalue threshold (i.e. 0.05). Moreover, the Pearson's correlation coefficient score between the predicted and true GDT-TS scores on the testing data set is 0.913, indicating that the predicted and true scores are highly linearly correlated. The results demonstrate that integrating alignment e-value with other features by SVM can improve the accuracy of ranking models over the naïve e-value based model ranking method and a state-of-art-black-box model evaluation method (i.e. APOLLO).

### Evaluation of the multiple-alignment based SVM model quality assessment method

The global average RMSE and ABS of the SVMtrained with the best set of parameter values (w, c, g)=(0.1, 2.0, 0.05) on the ten-fold training data set were 0.185 and 0.149, respectively. The trained SVM predictor was applied to predict the GDT-TS scores of models of 47 CASP9 targets generated from 3809 multiple protein sequence alignments that were not used in training. The RMSE and ABS were respectively, 0.176 and 0.142. This error is higher than that of the pairwise alignment-based predictor tested on models generated from PSI-BLAST alignments alone in the previous experiment, probably due to the higher diversity in alignments and model quality in this experiment. However, the advantage of this SVM predictor is that it can be applied to the alignments generated from any alignment methods and does not require an alignment e-value as input, which varies from one alignment method to another. The RMSE and ABS of the trained SVM predictor with the best parameter values on each fold of the training data, as well as the test data set are shown in Table 3.

We also used the predicted model quality scores to rank the models of 47 CASP9 targets in the testing data [11]. The total real GDT-TS score of the top 1 models selected by the multiple alignment based SVM predictor for these targets was compared with that of the top 1 models selected by APOLLO. The total GDT-TS score of the top 1 models selected by the multiple-alignment based SVM predictor is 22.59, which is lower than 25.26 of APOLLO. The lower performance of this multiple sequence alignment based SVM predictor is probably due to the lack of the alignment e-value feature used in the pairwise alignment based SVM predictor. Thus, one direction of improving multiple sequence alignment-based method is to include some features similar to the e-value of measuring the significance of alignments. And despite the lower performance of the current implementation of the multiple sequence alignment based SVM predictor, it is likely complementary with the black-box model quality assessment methods like APOLLO, because it used completely different features in prediction. And compared to the pairwise model comparison method like APOLLO that needs a pool of models of a protein as input, the alignment-based model quality assessment methods can be applied to assess the quality of one single model.

Furthermore, the Pearson's correlation coefficient score between the predicted and true GDT-TS scores on the testing data set is 0.969, indicating that the predicted and true model quality scores are highly linearly correlated.]

## Conclusions

In this work, we designed and developed a SVM protein model quality prediction method, taking either a pairwise sequence alignment or a multiple-sequence alignment as input. The evaluation results showed that integrating pure sequence alignment features with a SVM is an effective approach to protein model quality assessment. The new method can be integrated with template-based protein modelling methods to rank and select models. Since user-friendly and publicly accessible web-servers are important for making bioinformatics methods available to the community [25], we will make the model quality assessment methods developed in this work available as an easy-to-use web service for the community in the future.

## Figures and Tables

**Table 1:** The RMSE and ABS of the pairwise sequence alignment based SVM with the best parameter set on each fold of the raining data as well as the testing data set.

The data set	RMSE	ABS
Fold 1 of the training data	0.0868	0.0606
Fold 2 of the training data	0.0923	0.0674
Fold 3 of the training data	0.0821	0.0631
Fold 4 of the training data	0.0771	0.0557
Fold 5 of the training data	0.0783	0.0566
Test data	0.0978	0.0734

**Table 2:** The p-values on the scores of our SVM predictor and the e-value based model selection method and on the scores of our SVM predictor and the APOLLO based on t-test and Wilcox-test.

Methods	P-value (t-test)	P-value (Wilcox-test)
SVM predictor VS e-value based method	0.044	0.042
SVM predictor VS APOLLO	0.044	0.016

**Table 3:** The RMSE and ABS of the trained multiple sequence alignment based SVM with the best parameter set on each fold of the training data as well as the test data set.

The data set	RMSE	ABS
Fold 1 of the training data	0.2057	0.1678
Fold 2 of the training data	0.1516	0.1238
Fold 3 of the training data	0.1746	0.1393
Fold 4 of the training data	0.1538	0.1226
Fold 5 of the training data	0.1677	0.1383
Fold 6 of the training data	0.1692	0.1348
Fold 7 of the training data	0.1900	0.1487
Fold 8 of the training data	0.2330	0.1873
Fold 9 of the training data	0.2287	0.1939
Fold 10 of the training data	0.1721	0.1377
Test data	0.1764	0.1423

## References

[1] Schnell JR, Chou JJ (2008). Structure and mechanism of the M2 proton channel of infuenza A virus. Nature.

[2] Berardi MJ, Shih WM, Harrison SC, Chou JJ (2011). Mitochondrial uncoupling protein 2 structure determined by NMR molecular fragment searching. Nature.

[3] OuYang B, Xie S, Berardi MJ, Zhao X, Dev J (2013). Unusual architecture of the p7 channel from hepatitis C virus. Nature.

[4] Chou KC (2004). Structural bioinformatics and its impact to biomedical science. Curr Med Chem.

[5] Chou KC (2005). Coupling interaction between thromboxane A2 receptor and alpha-13 subunit of guanine nucleotide-binding protein. J Proteome Res.

[6] Lundström J, Rychlewski L, Bujnicki J, Elofsson A (2001). Pcons: A neural-network-based consensus predictor that improves fold recognition. Protein Sci.

[7] Wang Z, Tegge AN, Cheng J (2009). Evaluating the absolute quality of a single protein model using structural features and support vector machines. Proteins.

[8] Wang Z, Eickholt J, Cheng J (2011). APOLLO: A quality assessment service for single and multiple protein models. Bioinformatics.

[9] Benkert P, Tosatto SC, Schomburg D (2008). QMEAN: A comprehensive scoring function for model quality assessment. Proteins.

[10] Chen H, Kihara D (2008). Estimating quality of template-based protein models by alignment stability. Proteins.

[11] Li J, Deng X, Eickholt J, Cheng J (2013). Designing and benchmarking the MULTICOM protein structure prediction system. BMC Struct Biol.

[12] Cheng J, Li J, Wang Z, Eickholt J, Deng X (2012). The MULTICOM toolbox for protein structure prediction. BMC Bioinformatics.

[13] Cortes C, Vapnik V (1995). Support-vector networks. Machine Learning.

[14] Henikoff S, Henikoff JG (1992). Amino acid substitution matrices from protein blocks. Proc Natl Acad Sci U S A.

[15] Moult J, Fidelis K, Kryshtafovych A, Tramontano A (2011). Critical assessment of methods of protein structure prediction (CASP)--round IX. Proteins.

[16] Altschul SF, Madden TL, Schäffer AA, Zhang J, Zhang Z (1997). Gapped BLAST and PSI-BLAST: A new generation of protein database search programs. Nucleic Acids Res.

[17] Contreras-Moreira B, Ezkurdia I, Tress ML, Valencia A (2005). Empirical limits for template-based protein structure prediction: the CASP5 example. FEBS Lett.

[18] Zhang Y, Skolnick J (2004). Scoring function for automated assessment of protein structure template quality. Proteins.

[19] Joachims T (1999). Making large scale SVM learning practical.

[20] Chou KC, Zhang CT (1995). Prediction of protein structural classes. Crit Rev Biochem Mol Biol.

[21] Gonnet GH, Cohen MA, Benner SA (1992). Exhaustive matching of the entire protein sequence database. Science.

[22] Söding J, Biegert A, Lupas AN (2005). The HHpred interactive server for protein homology detection and structure prediction. Nucleic Acids Res.

[23] Li H, Handsaker B, Wysoker A, Fennell T, Ruan J (2009). The Sequence Alignment/Map format and SAMtools. Bioinformatics.

[24] Zhou H, Zhou Y (2005). SPEM: Improving multiple sequence alignment with sequence profiles and predicted secondary structures. Bioinformatics.

[25] Chou KC, Shen HB (2009). Review: Recent advances in developing webservers for predicting protein attributes. Natural Sci.

